# Children’s Eyes

**Published:** 1877-10

**Authors:** 


### The Bistoury.

ELMIRA, N. Y., OCTOBER, 1877.

The BISTOURY is published Quarterly, upon the 1st of April, July, October and January, at Fifty Cents a year in advance.

### CHILDREN'S EYES.

Our attention has been recently called to the prevalence of imperfect vision among school children, investigation proving that such defects are more numerous than we are wont to suppose. For a number of years it has been our duty to visit frequently our grammar schools, when our attention has now and then been called to the inability of certain scholars to recite well in our presence, when at other times they have been the best prepared and most apt in their respective classes.

Examination of their eyes proved them to be suffering from defective vision, caused from near-sightedness, oversightedness, or other anomalies of refraction.

The condition of the eyes known as *hyperopia*, in which the eyeball is flattened from before backwards, is a very frequent defect in the eyes of school children, giving rise to serious diminution of vision, particularly under excitement, as when reciting in the presence of strangers. The front to rear axis of such eyes being too short, the refractive power is not sufficient to bring the parallel rays of light to a focus upon the retina, but is adapted for convergent rays alone, consequently, the child is only able to focus such rays as are first rendered convergent by being passed through a convex lens. A child with such eyes, by a great effort at accommodation, can read the lesson upon the black board, but will complain of headache by reason of such effort. When reciting before visitors, or upon dark days, when the light is insufficient, he will hesitate, stammer and blunder through his lesson, be accused of stupidity and sent in disgrace to his seat, when the truth is, the poor child was really unable to read the print, by reason of the disturbance to his nervous system which lessened his ability to accommodate his eyes to the print. If such a child is "forced" in the school room, and has an ambition to keep up with his class, it will be soon discovered that his eyes turn towards the nose, when he is said to be cross-eyed. This circumstance is owing to the excessive use of the muscles which turn the eyes inward, in the endeavor to increase the power of accommodating the eye to short distances, as in the act of reading.

So frequent is this defect, that there is not a school room in our city, containing thirty-five pupils, that do not have among their number from one to five squinting eyes!

And this is but one defect of the eyes. How many more may be found upon examination to be suffering from near-sightedness, and other difficulties of vision, will perhaps never be known, until some measures are adopted by our school Boards to have suitable investigations made upon a matter of so much importance to our children.

Ophthalmologists know from experience, that instances are by no means rare where glasses are found to be necessary among children six or eight years of age, and that not infrequently, small children, when accidentally putting on their grand parents' spectacles, have found a new and brighter world opened for their inspection and delight.

The use of glasses is often objected to by parents or friends, supposing that the eyes will become weakened through their use, or that the trouble will be remedied later in life. Nothing could be further from the truth. Near-sighted or oversighted eyes should be promptly aided by suitable glasses, fitted by a competent person, and the longer their use is delayed, the weaker the eyes become. This fact holds good with the eyes of both the young and old. If you have any reason to suspect defects in the vision of your school children,—as may be manifested in not being able to read the lessons upon the black board readily, or in holding the book too close to the face, in reading, do not delay in consulting competent authority upon the subject.

